# 26 Opioid Prescription in Burns: A Large Database Analysis from 1990 to 2021

**DOI:** 10.1093/jbcr/irac012.029

**Published:** 2022-03-23

**Authors:** Elvia L Villarreal, Steven E Wolf, George Golovko, Kendall Wermine, Sunny Gotewal, Lyndon G Huang, Kassandra K Corona, Shelby P Bagby, Phillip H Keys, Alejandro A Joglar, Giovanna De La Tejera, Shivan N Chokshi, Juquan Song, Amina El Ayadi

**Affiliations:** University of Texas Medical Branch, Galveston, Texas; University of Texas Medical Branch at Galveston, Galveston, Texas; University of Texas Medical Branch at Galveston, Galveston, Texas; University of Texas Medical Branch, Galveston, Texas; University of Texas Medical Branch, Galveston, Texas; University of Texas Medical Branch, Galveston, Texas; University of Texas Medical Branch, Galveston, Texas; University of Texas Medical Branch, Galveston, Texas; University of Texas Medical Branch, Galveston, Texas; University of Texas Medical Branch, Galveston, Texas; University of Texas Medical Branch, Galveston, Texas; University of Texas Medical Branch at Galveston, Galveston, Texas; University of Texas Medical Branch, Galveston, Texas; University of Texas Medical Branch at Galveston, Galveston, Texas

## Abstract

**Introduction:**

The use of opioids in the medical field has contributed to the growing opioid epidemic. Nonetheless, opioids remain imperative in the treatment for pain management in burns. While some studies have addressed the use of opioids in surgery, a comprehensive analysis of the pattern of opioids use in burns has not been investigated. This study aims to identify trends of opioid use and investigate the risk of opioid related disorders in burn patients.

**Methods:**

Data was obtained from TriNetX, a national research database that provides medical records of de-identified patients. The study population includes patients that were prescribed an opioid, ICD-10 code CN101, on or after any instance of burn between January 1^st^, 1990 and September 19^th^, 2021. Patient population was further stratified by the decade in which patients received opioids for pain following burn injury: 1990-1999, 2000-2009, 2010-2019, and 2020-September 19^th^, 2021. Five outcomes were investigated: opioid related disorders, opioid dependence, opioid abuse, intentional self-harm, and mental and behavioral disorders due to psychoactive substance use. Cohorts were matched for age at index, sex, and race. Statistical analysis used risk ratios with a 95% confidence interval, and p< 0.05 was considered significant.

**Results:**

We identified 8,421 patients that were prescribed an opioid between 1990-1999, 30,846 patients from 2000-2009, 169,991 patients from 2010-2019, and 30,966 patients from 2020-present. When compared to the 2000s cohorts, the 1990s patients had a 47% decrease in risk of opioid related disorders, with a 53% decrease in risk of opioid dependence, 45% decrease in risk in opioid abuse, 11% decrease in risk of mental and behavioral disorders due to psychoactive substance use, and 63% reduced risk of intentional self-harm.

Comparison of the 2000-2009 to 2010-2019 cohorts showed increased risk of opioid related disorders (RR= 1.912), opioid dependence (RR=1.569), opioid abuse (RR=1.677), mental and behavioral disorders (RR =1.733), and intentional self-harm (RR=2.027). When compared to 2020-present, the 2010-2019 patient cohort had 10 times the risk of developing opioid-related disorders, with 3 times the risk for opioid dependence and behavioral disorders, and 5 times the risk for opioid abuse and intentional self-harm.

**Conclusions:**

The risk of opioid related disorders in the 1990s was lower compared to the 2000s. Since 2000, the risk of opioid related disorders has significantly increased. Recognizing the risks of opioid prescriptions in burn patients is imperative when addressing the role of physicians in controlling the constantly growing opioid epidemic.

